# Hyaluronan abrogates imatinib-induced senescence in chronic myeloid leukemia cell lines

**DOI:** 10.1038/s41598-019-47248-8

**Published:** 2019-07-29

**Authors:** Silvina Lompardía, Mariángeles Díaz, Matías Pibuel, Daniela Papademetrio, Daniela Poodts, Cintia Mihalez, Élida Álvarez, Silvia Hajos

**Affiliations:** 1 Universidad de Buenos Aires. Facultad de Farmacia y Bioquímica. Departamento de Microbiología, Inmunología y Biotecnología, Cátedra de Inmunología, Buenos Aires, Argentina; 2 Universidad de Buenos Aires, CONICET, Instituto de Estudios de la Inmunidad Humoral (IDEHU), Buenos Aires, Argentina

**Keywords:** Senescence, Cancer therapy, Chronic myeloid leukaemia

## Abstract

Hyaluronan (HA) is the main glycosaminoglycan of the extracellular matrix. CD44 is the most important HA receptor, and both have been associated with poor prognosis in cancer. Chronic myeloid leukemia (CML) is characterized by the presence of a constitutively activated tyrosine kinase (Breakpoint Cluster Region - Abelson murine leukemia viral oncogene homolog1, BCR-ABL). It is mainly treated with BCR-ABL inhibitors, such as imatinib. However, the selection of resistant cells leads to treatment failure. The aim of this work was to determine the capacity of HA (high molecular weight) to counteract the effect of imatinib in human CML cell lines (K562 and Kv562). We demonstrated that imatinib decreased HA levels and the surface expression of CD44 in both cell lines. Furthermore, HA abrogated the anti-proliferative and pro-senescent effect of Imatinib without modifying the imatinib-induced apoptosis. Moreover, the inhibition of HA synthesis with 4-methylumbelliferone enhanced the anti-proliferative effect of imatinib. These results suggest that Imatinib-induced senescence would depend on the reduction in HA levels, describing, for the first time, the role of HA in the development of resistance to imatinib. These findings show that low levels of HA are crucial for an effective therapy with imatinib in CML.

## Introduction

It is known that the tumor microenvironment characteristics are crucial for the progression of several malignancies^1^. One of the main components of extracellular matrix is hyaluronan (HA), a lineal non-sulfated glycosaminoglycan consisting of repetitive units of D-glucuronic acid and N-acetyl-D-glucosamine. HA plays many physiological roles, such as the organization and maintenance of the tissue architecture, wound healing, leukocyte trafficking, and cell growth and differentiation, among others. HA also seems to play a role in stem cells niches where it would protect these cells from DNA damage. This function is accomplished through its antioxidant action and the activation of efflux pumps that extrude genotoxic compounds^2,3^.

The amount of HA in a tissue results from a complex balance between its synthesis by glycosyltransferases (hyaluronan synthases, HAS), its internalization via surface receptors and its degradation mediated by hyaluronidases (HYALs)^2,4–6^. Changes occurring in any of these processes lead to an imbalance in the quantity and quality of HA. It has been demonstrated that low HA levels in bone marrow inhibit the capacity of the cellular microenvironment to maintain a normal hematopoiesis^7,8^. In several solid tumors, the amount of HA exceeds the physiological levels enhancing cell proliferation, migration, apoptosis evasion and multidrug resistance (MDR)^9–13^. These effects are exerted through the activation of several signaling pathways, such as phosphoinositide 3-kinase (PI3K)/protein kinase B (Akt) and Mitogen-Activated Protein Kinases (MAPK), as a result from the interaction of HA with its receptors, being CD44 the most studied one in oncology^14^.

However, little is it known about the role of HA on chronic myeloid leukemia (CML) progression. CML is a myeloproliferative syndrome characterized by the presence of the Philadelphia chromosome (Ph+), which encodes a constitutively activated tyrosine kinase (Breakpoint Cluster Region - Abelson murine leukemia viral oncogene homolog 1, BCR-ABL)^15–17^. The first line treatment for CML consists of BCR-ABL inhibitors such as imatinib, nilotinib and dasatinib^15,16^. Although imatinib is a highly effective drug for the treatment of CML, drug resistance may occur, leading to therapeutic failure. The identification of the molecules involved in resistance phenomena is then of great value for the development of novel effective chemotherapeutic agents.

It has recently been demonstrated that low levels of CD44 are associated with a better therapeutic response to nilotinib in different models of CML^18^. We have previously demonstrated that human CML cell lines synthesize HA that is crucial to prevent cells from undergoing senescence and resist the cytostatic effect of vincristine^19^. In addition, we have shown that HA oligomers and 4-methylumbelliferone (4MU) enhance the effect of imatinib on CML cell lines^20,21^. Due to their small size, HA oligomers bind HA receptors without activating them^10^, while 4MU inhibits the HA synthesis by depleting the levels of UDP-glucuronic acid (substrate of HAS)^22^.

One of the most commonly used *in vitro* CML models is the K562 human cell line^23,24^. In these cells, the anti-proliferative effect of imatinib is mediated by the induction of apoptosis and senescence^21,25^. These biological processes are two of the most important mechanisms of tumor suppression. Apoptosis is a type of programmed cell death^26^, while senescence is a terminal differentiation stage characterized by an irreversible cell cycle arrest^27–31^.

Multiple factors are known to contribute to the development of chemoresistance, being the extracellular matrix a key component of the tumor microenvironment. We hypothesize that the HA present in such microenvironment enhances MDR favoring leukemia progression. The aim of this work was to determine whether high molecular weight HA abrogates the effect of imatinib in human CML cell lines, describing for the first time the role of HA on imatinib resistance. The findings presented herein highlight the importance of reducing the levels of HA for an effective therapy with imatinib in CML.

## Results

### Imatinib reduces BCR-ABL and HA levels, as well as CD44 surface expression

The capacity of imatinib to modulate BCR-ABL, HA and CD44 levels was first analyzed. BCR-ABL levels were evaluated by western blot (WB), HA levels were analyzed by ELISA and the expression of CD44 by flow cytometry (FC).

**Figure 1:**
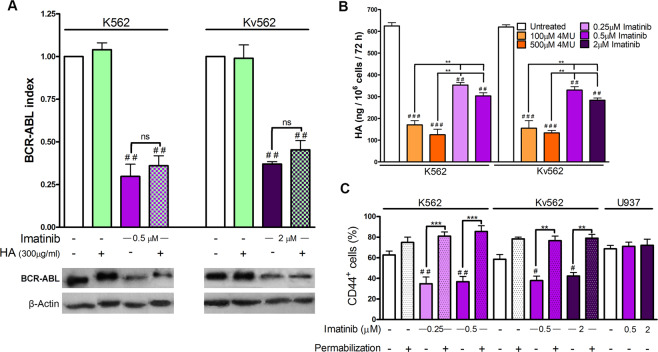
Effect of imatinib on BCR-ABL, HA and CD44 levels. (**A**) K562 and Kv562 cells were treated either with imatinib, HA (high molecular weight) or a combination of both for 24 h. Expression levels of BCR-ABL were evaluated by WB. Results are expressed as: BCR-ABL index = (BCR-ABL/β-actin)_treated_/(BCR-ABL /β-actin)_untreated_. Data are expressed as the mean ± SEM of at least three independent experiments ^##^p < 0.01 treated *vs*. untreated cells, ns = non-significant difference (p > 0.05) between the treatments indicated. (**B**) Levels of HA in culture supernatant were evaluated by ELISA after the treatment with either imatinib or 4MU for 72 h. Values are expressed as the mean ± SEM (ng HA/10^6^cells/72 h) of at least three independent experiments. ^###^p < 0.001 and ^##^p < 0.01, treated *vs*. untreated cells and ^**^p < 0.01 between the treatments indicated. (**C**) Both cell lines were treated with imatinib for 24 h and the CD44 expression (with or without permeabilization) was then evaluated by FC. U937 cells were used as BCR-ABL negative control. Results are expressed as mean ± SEM of CD44 positive cells (%) of at least three independent experiments. ^##^p < 0.01, ^#^p < 0.01, treated *vs*. untreated cells and ***p < 0.001, **p < 0.01 between the treatments indicated.

Figure 1A shows that HA did not modify the expression of BCR-ABL, while imatinib decreased the expression levels with respect to the baseline condition in K562 and Kv562 cells. Moreover, in cells co-treated with imatinib and HA, the levels of BCR-ABL were similar to those obtained with imatinib alone. Figure 1B shows that HA levels in the culture supernatant of imatinib-treated cells were diminished, as compared to untreated control cells. However, such decrement was of a smaller magnitude than the one obtained with 4MU. It is noteworthy that we have previously demonstrated that 4MU completely inhibits the synthesis of HA^19^. Figure 1C shows that the treatment with imatinib decreased the surface expression of CD44 in both cell lines without modifying the total expression levels of this marker, suggesting that this drug induces the internalization of this receptor. The U937 cell line was used as a negative control for BCR-ABL and a positive control for CD44^32,33^.

Thus, imatinib reduced HA levels and the surface expression of CD44, suggesting that BCR-ABL would favor the accumulation of HA in the culture supernatant and of CD44 on the cell surface.

### HA abrogates the anti-proliferative effect of imatinib without modifying imatinib-induced apoptosis

Considering that imatinib induced a partial decrease of HA levels, it was interesting to evaluate if such reduction was necessary for the drug to exert its anti-proliferative and pro-apoptotic effects. For that purpose, both cell lines were treated with either imatinib, HA or a combination of both to determine cell proliferation rates by the tritiated thymidine ([^3^H]TdR) uptake assay and the apoptosis levels by the DNA fragmentation, annexin-PE/7AAD and subG1 peak assays.

**Figure 2:**
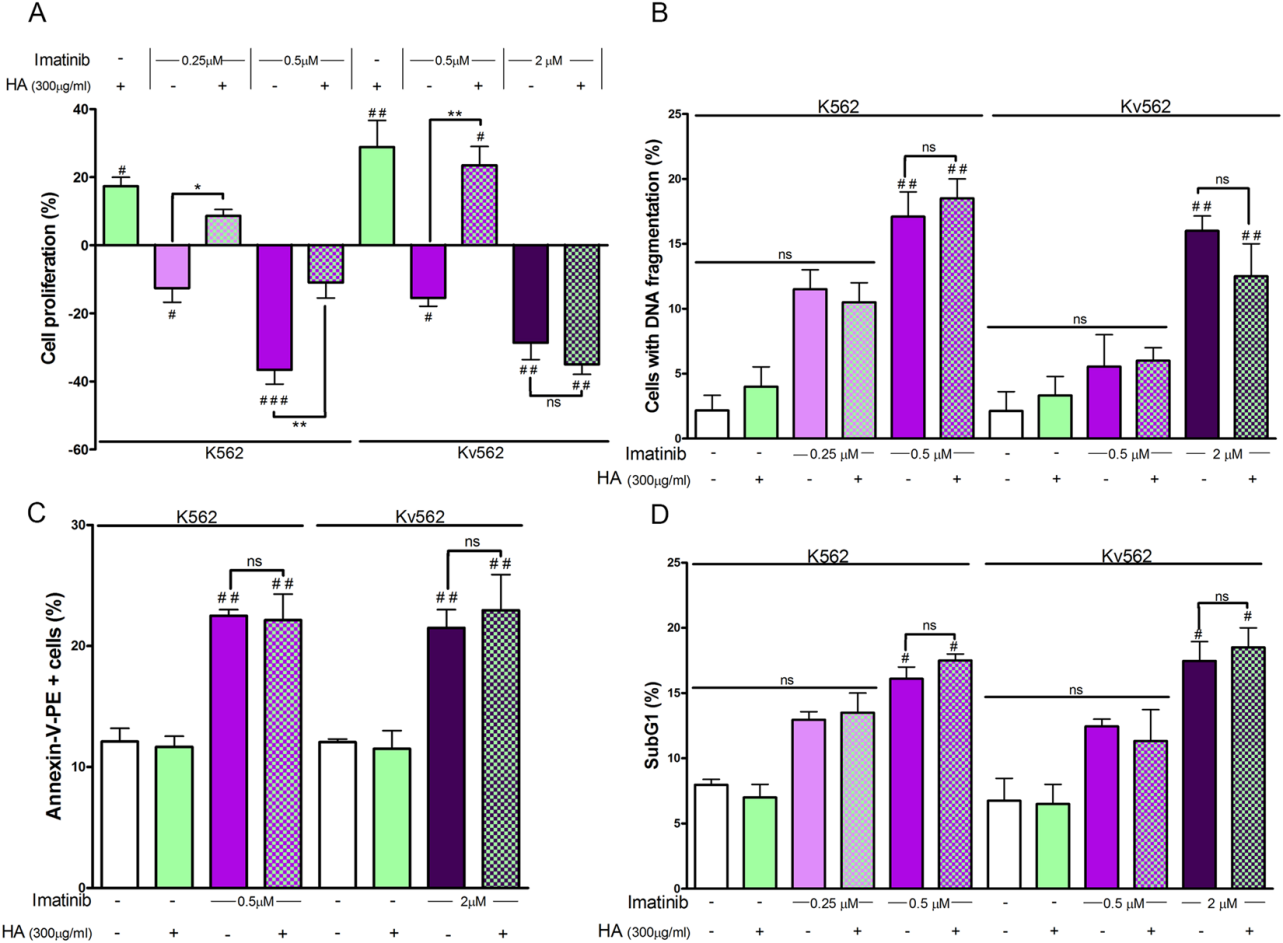
Evaluation of cell growth and apoptosis. (**A**) Both cell lines were treated with either HA (high molecular weight), imatinib or a combination of both. Cell growth was determined by the [^3^H]TdR uptake assay after 48 h. Results are expressed as: Cell proliferation (%) = (treated cpm × 100/untreated cpm) − 100. Results are expressed as means ± SEM of at least five independent experiments ^###^p < 0.01, ^##^p < 0.01 and ^#^p < 0.05, treated *vs*. untreated cells, **p < 0.01, *p < 0.05 and ns = non-significant (p > 0.05) between the treatments indicated. To determine if the co-treatment of HA and imatinib abrogates the induction of apoptosis, both cell lines were treated with these compounds alone or in combination for 48 h and then three different parameters were analyzed: (**B**) DNA fragmentation was analyzed by fluorescence microscopy, for which K562 and Kv562 cells were fixed with PFA and stained with DAPI. (**C**) Membrane asymmetry was evaluated by FC, for which cells were stained with annexinV-PE/7AAD after each treatment. (**D**) The hypodiploid content was evaluated by FC. For this experiment, cells were fixed with ethanol and stained with DAPI. Results are expressed as means ± SEM of three independent experiments ^##^p < 0.01 treated *vs*. untreated cells, ^#^p < 0.05 treated *vs*. untreated cells, while ns = non-significant (p > 0.05) between the treatments indicated.

Figure 2A shows that, unlike imatinib, the treatment with HA enhanced the proliferative capacity of both cell lines. HA counteracted the anti-proliferative effect of imatinib on K562 cells, at all the drug doses tested. However, in Kv562 cells, this phenomenon was only observed with the combination of HA with the lowest dose of imatinib (0.5 µM).

It was also interesting to evaluate the effect of HA on imatinib-induced apoptosis in both cell lines. HA modified neither the percentage of DNA fragmentation (Fig. 2B), nor the number of annexin-PE positive cells (Fig. 2C), nor the subG1 peak (Fig. 2D), as compared to control cells. Conversely, the treatment with 0.5 and 2 µM imatinib increased such parameters in K562 and Kv562 cells, respectively. However, the co-treatment of cells with HA and imatinib did not modify the apoptosis levels, as compared with imatinib alone in both cell lines.

These results suggest that HA would abrogate part of the therapeutic effect of imatinib by suppressing its anti-proliferative effect. However, HA does not seem to be involved in the mechanism of evasion from imatinib-induced apoptosis.

### HA prevents imatinib-induced senescence

We have previously described that HA prevents the induction of senescence in both cell lines^19^ and that it suppresses the anti-proliferative effect of imatinib. We then evaluated if HA was able to abrogate imatinib-induced senescence. To that end, the activity of senescence-associated β-galactosidase (SA-β-gal) and the presence of senescence-associated heterochromatin foci (SAHF) were determined.

**Figure 3:**
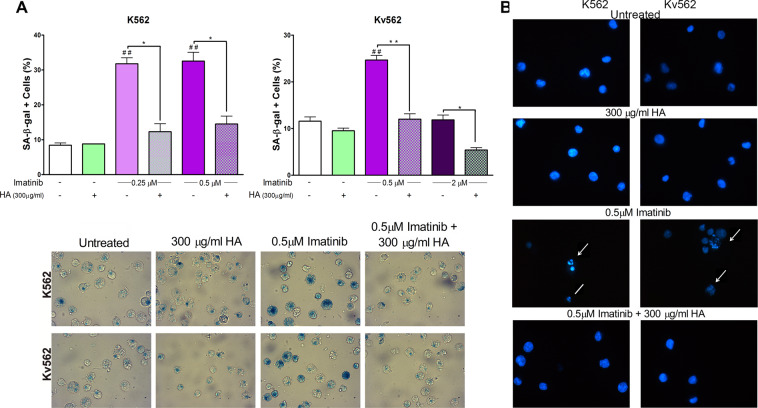
Evaluation of senescence. To determine if the combination of HA (high molecular weight) and imatinib prevents the induction of senescence, both cell lines were treated with either each compound alone or with a combination of both for 48 h. SA-β-gal and SAHF were then evaluated. (**A**) SA-β-gal activity (upper panel). Results are expressed as means ± SEM of three independent experiments ^##^p < 0.01 treated *vs*. untreated cells, while **p < 0.01 and *p < 0.05 between the treatments indicated (lower panel). Pictures are from a representative experiment (**B**) SAHF, magnification 400X. Arrows indicate nuclei with SAHF, while lines indicate nuclei with DNA fragmentation, which are characteristic of senescent and apoptotic cells, respectively.

As shown in Fig. 3, HA modified neither the percentage of SA-β-gal positive cells nor the presence of SAHF with respect to baseline conditions in both cell lines. Contrarily, imatinib at 0.25 and 0.5 µM increased such parameters in both cell lines. However, the treatment with 2 µM imatinib did not induce senescence in Kv562 cells. For all doses of imatinib that induced senescence, the addition of HA prevented the appearance of SA-β-gal positive cells (Fig. 3A) and SAHF (Fig. 3B), thus restoring the baseline conditions.

These results suggest that HA abrogates imatinib-induced senescence and that HA would be involved in the development of imatinib resistance.

### HA abrogates the effect of imatinib on the pAkt/Akt ratio but not on the pERK/ERK ratio

Knowing that the PI3K/Akt and MAPK pathways are crucial for leukemic cell growth^34^ and that they are modulated by HA, it was interesting to assess if such signaling pathways participate in the effects described above. Therefore, WB assays were performed to determine pAkt/Akt and pERK/ERK ratios after the treatment with either HA, Imatinib or combinations of them.

**Figure 4:**
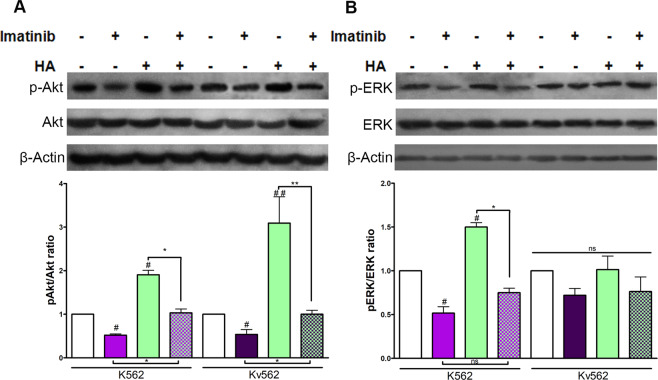
Modulation of pAkt/Akt and pERK/ERK ratios. (**A**) K562 and Kv562 cells were treated with either HA (high molecular weight), Imatinib (0.5 µM for K562 cells and 2 µM for Kv562 cells) or a combination of both for 24 h. The phosphorylation of Akt was evaluated by WB. Results are expressed as: pAkt/Akt ratio = [(pAkt/β-actin)/(Akt/β-actin)]_treated_/[(pAkt/β-actin)/(Akt/β-actin)]_untreated_. Results are expressed as means ± SEM of at least three independent experiments ^##^p < 0.01, ^#^p < 0.05 treated *vs*. untreated cells and ** p < 0.01, *p < 0.05 between the treatments indicated. (**B**) Both cell lines were treated with either HA (high molecular weight), imatinib (0.5 µM for K562 cells and 2 µM for Kv562 cells) or a combination of both for 24 h. The phosphorylation of ERK was evaluated by WB. Results are expressed as pERK/ERK ratios. Results are expressed as means ± SEM of at least three independent experiments, ^#^p < 0.05 treated *vs*. untreated cells, while *p < 0.05 and ns = no significant (p > 0.05) between the treatments indicated.

Figure 4A shows that HA increased the pAkt/Akt ratio in both cell lines, while this ratio was found to be decreased upon treatment with imatinib. The co-treatment with HA and imatinib restored the baseline conditions in both K562 and Kv562 cells. The biological replicates are shown in Supplementary Fig. S1.

Figure 4B shows that HA augmented the pERK/ERK ratio while imatinib reduced this ratio only in K562 cells. However, the addition of HA did not inhibit the effect of imatinib on ERK phosphorylation. Moreover, in Kv562 cells, HA, imatinib, as well as the combination of both did not modify the pERK/ERK ratio with respect to the baseline conditions. The biological replicates are shown in Supplementary Fig. S1.

Considering these results, we suggest that the anti-proliferative and pro-senescent effect of imatinib is abrogated by HA through the modulation of the PI3K/Akt pathway.

### The inhibition of HA synthesis enhances the anti-proliferative and pro-senescent effect of imatinib

Taking into account that the combined treatment with 4MU and imatinib enhances the induction of senescence^20^, we determined if the reduction of HA levels induced by these drugs mediates those effects. For this purpose, both cell lines were treated with either HA, 4MU, imatinib or combinations of them. Cell growth, induction of senescence, and the pAkt/Akt ratio were evaluated. The 4MU concentration employed was 100 µM, since we had previously demonstrated that the anti-proliferative and pro-senescent effects achieved with this dose are fully abrogated by the addition of HA. Higher doses of 4MU proved to have effects that were independent of the inhibition of the HA synthesis^19^.

**Figure 5:**
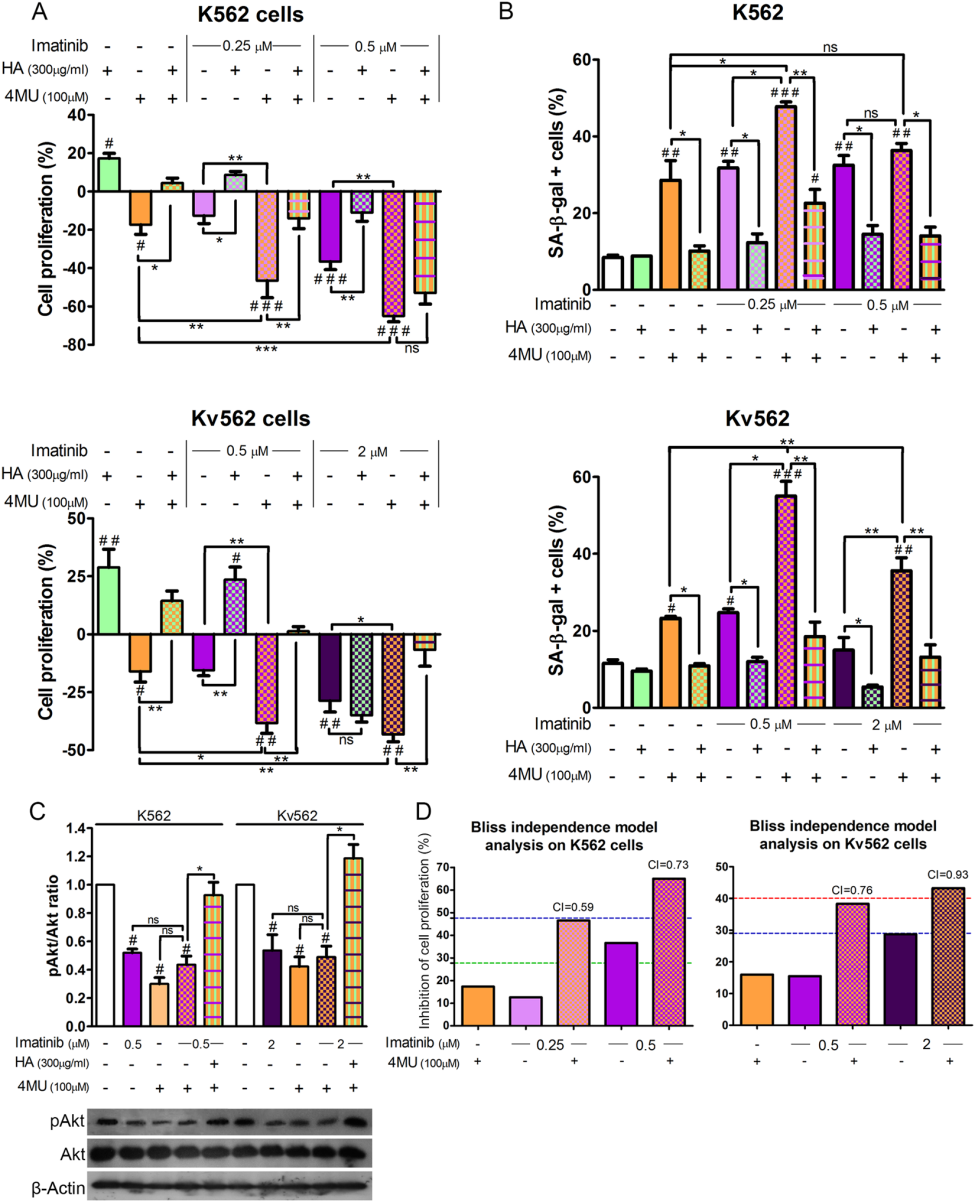
Effect of imatinib and the inhibition of HA synthesis on both cell lines. K562 and Kv562 cell lines were treated with either HA (high molecular weight), imatinib, 4MU or a combination of both. (**A**) Cell growth was determined by the [^3^H]TdR uptake assay for 48 h. Results are expressed as: Cell proliferation (%) = (treated cpm × 100/untreated cpm) − 100. Results are expressed means ± SEM of at least five independent experiments. (**B**) The induction of senescence was evaluated by the SA-β-gal activity assay. Results are expressed means ± SEM of at least three independent experiments. (**C**) The pAkt/Akt ratio was evaluated by WB. (**D**) For the inhibition of cell proliferation, the effect of 4MU and Imatinib combination was analyzed by effect-based strategy through Bliss independence model calculating the Combination Index (CI). Dotted lines represent the value of the expected additive effect calculated as: Expected additive effect = (4MU_effect_ + Imatinib_effect_ − 4MU_effect_ × Imatinib_effect_) × 100 (where 0 ≤ 4MU_effect_ ≤ 1 and 0 ≤ Imatinib_effect_ ≤ 1). The expected additive effect for 4MU + 0.25 µM Imatinib is represented by the green line, for 4MU + 0.5uM Imatinib is represented by the blue line and for 4MU + 2uM Imatinib is represented by the red line. The resulting CI was calculated as: CI = (4MU_effect_ + Imatinib_effect_ − 4MU_effect_ × Imatinib_effect_)/Combination_effect_. (where 0 ≤ 4MU_effect_ ≤ 1; 0 ≤ Imatinib_effect_ ≤ 1 and 0 ≤ Combination_effect_ ≤ 1), being CI < 1 synergism, CI = 1 additive and CI > 1 antagonism. All results are expressed as means ± SEM of three independent experiments. ^###^p < 0.01, ^##^p < 0.01 and ^#^p < 0.05 treated *vs*. untreated cells, while ***p < 0.001, **p < 0.01, *p < 0.05 and ns = non-significant (p > 0.05) between the treatments indicated.

As shown in Fig. 5A, the addition of HA to cells treated with 4MU and imatinib restored cell proliferation to baseline levels in all cases, except for K562 cells treated with 0.5 µM imatinib, 4MU and HA, in which the difference was not statistically significant with respect to cells co-treated with 4MU and imatinib.

Figure 5B shows that the addition of HA to cells treated with imatinib and 4MU decreased the percentage SA-β-gal K562 and Kv562 positive cells. In K562 cells, only the co-treatment with 0.5 µM imatinib and 4MU induced levels of senescence (percentage of SA-β-gal positive cells) that were similar to the percentage obtained with each inhibitor alone. However, the addition of HA to cells treated with imatinib and 4MU reduced this percentage to baseline levels. Similar results were obtained for the evaluation of SAHF (see Supplementary Fig. S2).

The evaluation of the pAkt/Akt ratio (Fig. 5C) demonstrated that imatinib, 4MU and the combination of both decreased Akt phosphorylation to similar levels, while the addition of HA to cells treated with imatinib and 4MU restored the baseline phosphorylation levels in both cell lines.

Finally, Fig. 5D shows that the combination of 4MU and imatinib had a synergistic effect in all doses tested at the level of the inhibition of cell proliferation since all combination indexes (CI) were less than 1.

These results indicate that the inhibition of HA synthesis enhances the anti-proliferative and pro-senescent effects of imatinib.

## Discussion

CML is a myeloproliferative syndrome whose first line treatment consists on BCR-ABL inhibitors such as imatinib, nilotinib and dasatinib. The adequate therapeutic management has significantly increased survival^35^. Imatinib is a highly effective drug; however, therapeutic failure is frequently observed due to the selection of resistant cells^36,37^. Therefore, identifying the molecules involved in imatinib resistance is crucial. In this work, we demonstrate for the first time that, on K562 and Kv562 CML cell lines, high molecular weight HA abrogates the anti-proliferative and pro-senescent effects of imatinib without modifying the apoptosis levels induced by this drug.

**Figure 6:**
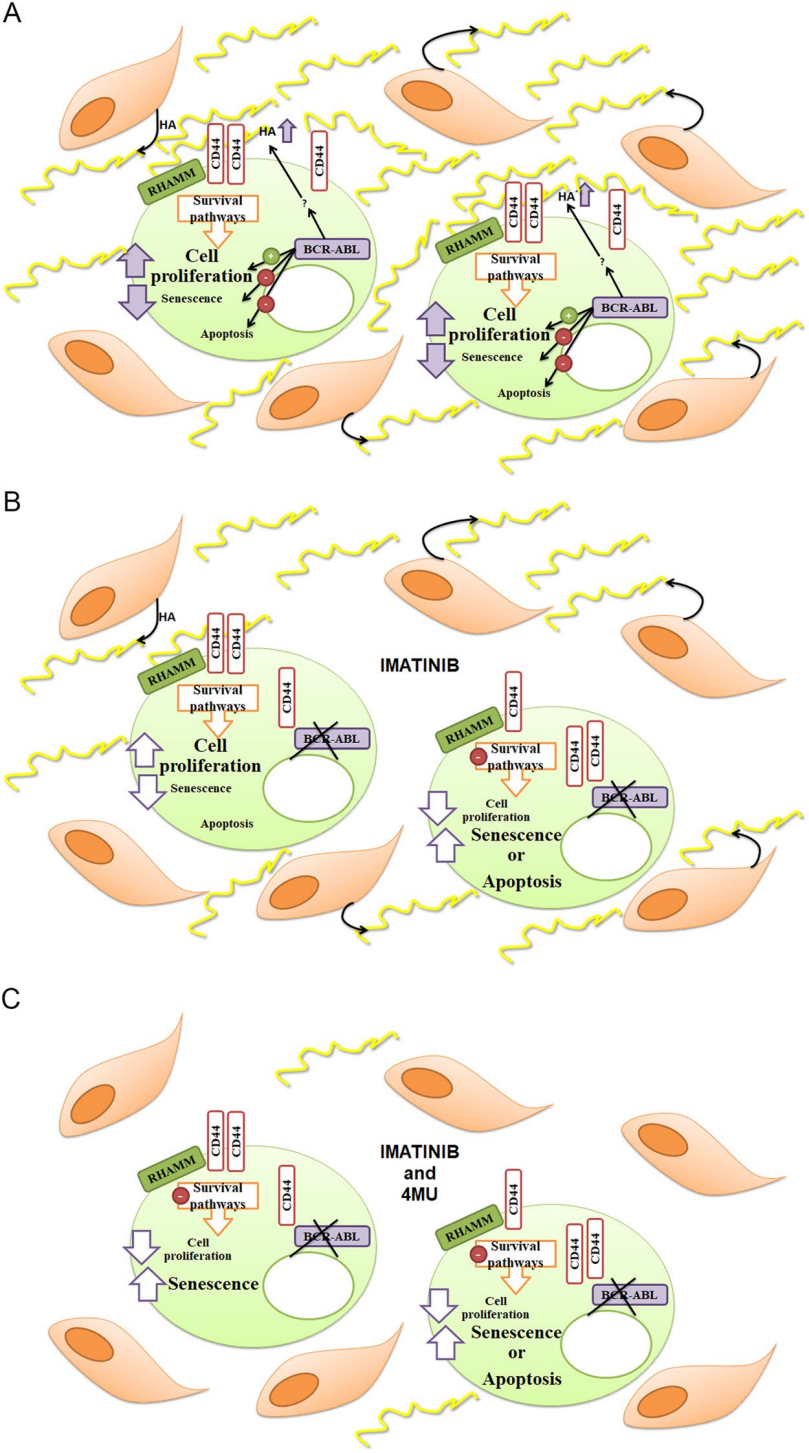
Suggested model explaining the involvement of HA in the therapeutic failure in CML. (**A**) Under pathophysiological conditions, CML cells would have survival signals triggered by BCR-ABL and HA. The latter would be synthesised by stromal bone marrow cells as well as by leukemic cells. BCR-ABL would favour the accumulation of HA in tumor microenvironment. (**B**) The inhibition of BCR-ABL by imatinib would decrease HA levels and the expression of CD44 on the cell surface. Imatinib abrogates cell proliferation favouring the induction of either senescence or apoptosis. The Imatinib-induction senescence would dependent on the reduction of HA levels. However, bone marrow stromal cells would synthesize HA, which could trigger the activation of survival pathways, thus being involved in the development of imatinib resistance. (**C**) The combination of imatinib and 4MU would prevent the accumulation of HA favouring the induction of senescence and apoptosis, thus allowing a better therapeutic response.

We first demonstrated that imatinib decreased BCR-ABL levels. This observation is in line with those of other authors, who described that imatinib induces the expression of miR-138 and miR-203, which inhibit BCR-ABL mRNA translation^38,39^. Furthermore, imatinib decreased the levels of HA and the expression of CD44 on the cell surface without modifying the total CD44 expression, suggesting that this receptor would be internalized. It is known that HYAL2, in collaboration with CD44, binds and degrades HA into smaller fragments on the cell surface. These fragments would then be engulfed and internalized in acidic vesicles and finally degraded by HYAL1^6,40^. Considering that imatinib reduces the CD44 surface expression (without modifying the total CD44 expression) and that it also decreases HA levels; it is proposed that this drug would enhance HA catabolism through the inhibition of BCR-ABL. Another mechanism that would explain the reduction of HA levels would be the inhibition of its synthesis; since the activation of the PI3K/Akt pathway enhanced HA synthesis and imatinib decreased the pAkt/Akt ratio. In addition, we suggest that BCR-ABL would favor the accumulation of HA and the expression of CD44, since they provide a proliferative advantage (Fig. 6A). Likewise, other authors have demonstrated that imatinib induces the degradation of the extracellular matrix, thus reducing the levels of collagen and heparan sulfate proteoglycans^41–43^. Furthermore, it has been demonstrated that imatinib reduces the production of HA in orbital tissue cultures^44^. Such effect would be mediated by the inhibition of platelet-derived growth factor receptor (PDGFR). It is noteworthy that the activation of this receptor in K562 cells leads to the differentiation to the megakaryocytic linage; being inactive under baseline conditions^45^. Recently Zhou H, *et al*. have reported that the inhibition of β-catenin and BCR-ABL decreases CD44 levels in K562 cells, in stem/progenitor cells in blastic crisis of CML samples as well as on *in vivo* models. The reduction of CD44 levels is crucial to achieve a better therapeutic response^18^. Besides, it has been reported that CD44 is a leukemic stem cell marker that is crucial for homing and cell proliferation^46^. Therefore, BCR-ABL is expected to promote the expression of CD44 on the cell surface, while the inhibition of BCR-ABL by imatinib leads to a reduction of CD44 levels on the cell surface.

We also showed that the addition of HA abrogated the anti-proliferative and pro-senescent effects of imatinib without modifying the inhibitor-dependent apoptosis in both cell lines. Therefore, the induction of senescence mediated by imatinib would depend on the reduction of HA levels. It is interesting that in Kv562 cells, 2 μM imatinib induced apoptosis, but did not induce senescence; and the addition of HA did not modify the anti-proliferative and pro-apoptotic effects of imatinib. Therefore, HA only prevented the induction of senescence and this effect was observed for all doses of imatinib which were able to induce senescence in both cell lines. Thus, BCR-ABL would induce the accumulation of HA, which favors cell growth and prevents the induction of senescence, one of the most important mechanisms of tumor suppression. The bone marrow presents high HA levels^7,8^. This microenvironment would trigger survival signaling pathways promoting imatinib resistance of CML cells. In addition, imatinib might reduce the accumulation of HA by Ph+ cells but could not modulate HA metabolisms in stromal cells (Fig. 6B). The reduction of HA levels would therefore improve the efficacy of imatinib therapy. Moreover, gelatinous transformation of the bone marrow has been described in patients with CML treated with imatinib as a late morphological change^47^. This transformation implies an increment in glycosaminoglycan levels, mainly HA, which would be involved in the therapeutic failure observed after a prolonged exposure to the drug.

The PI3K/Akt pathway is a pro-survival factor in leukemia stem cells and early committed leukemic precursors. The inhibition of this pathway constitutes a therapeutic approach^34^. We have previously demonstrated that HA activates PI3K in both cell lines (K562 and Kv562)^19^. In this work, we demonstrated that the addition of HA abrogates the reduction of the pAkt/Akt ratio induced by imatinib, thus restoring the baseline ratio. Therefore, HA would enhance the survival of these CML cell lines, generating imatinib resistance through the activation of PI3K. These results are in accordance with other report demonstrating that the activation of PI3K promotes imatinib resistance independently of BCR-ABL mutations^48^ and with our previous work showing that both HA oligomers and Ly294002 were able to sensitize K562 and Kv562 cells to the effect of imatinib by inhibiting the PI3K pathway^21^.

Similarly, the activation of MEK/ERK favors the development of resistance to imatinib, whereas the inhibition with UO126 favors the induction of apoptosis in K562 cells^49,50^. Nambu *et al*., 2010, have described that the BCR-ABL-independent activation of ERK favors imatinib resistance, demonstrating that imatinib decreases the pERK/ERK ratio in K562 cells but not in imatinib-resistant K562 cells, which do not have a mutated BCR-ABL^49^. The effect of imatinib on the pERK/ERK ratio in K562 is in agreement with our findings. However, Yu *et al*., 2002, have reported an increase in the pERK/ERK ratio exerted by imatininb^50^. The difference between this study and our findings can be attributed to the doses of imatinib employed and by the anti-pERK antibodies used in WB assays, which recognize different phosphorylation sites (we evaluated the phosphorylation on Tyr204 only, while You *et al*. evaluated Thr202 and Tyr204). Our results show that HA increased ERK phosphorylation, but it did not significantly counteract the effect of imatinib on pERK in K562 cells. On the other hand, neither HA nor imatinib nor their combination modulated ERK phosphorylation. Considering these results and those reported by Nambu *et al*. and Yu *et al*., the MAPK pathway would be also playing a role in the development of drug resistance in Kv562 cells.

We have previously demonstrated that 4MU and imatinib enhance the induction of senescence^20^. However, the mechanism exerted by 4MU to sensitize both cell lines to the effect of imatinib remained to be elucidated. In this work we demonstrated that the sensitization could be attributed to the inhibition of HA synthesis, since the addition of HA suppressed the effect exerted by the co-treatment of K562 and Kv562 cells with 4MU and imatinib. These results highlight the potential usefulness of 4MU as a co-adjuvant for the treatment of CML with imatinib. Moreover, in CML patients, 4MU would inhibit HA synthesis in both leukemic and stromal cells, improving the therapeutic response to imatinib (Fig. 6C). The microenvironment features are crucial for myeloid malignancies^51^, particularly, CML patients with bone marrow gelatinous transformation show high levels of HA and are prone to develop resistance to imatinib, therefore, 4MU could be a promising therapeutic alternative. In addition, the synergistic effect showed by the co-treatment is interesting from a therapeutic point of view, since the combination of both drugs would contribute to a common anti-proliferative effect through different sites of action. More studies are, however, needed to assert the beneficial effects of this therapy in humans.

In this work, we demonstrated for the first time that HA (high molecular weight) abrogated the anti-proliferative and pro-senescent effects of imatinib in K562 and Kv562 cells, suggesting an important role of HA in the development of imatinib resistance. Moreover, we hypothesize that the inhibition of BCR-ABL leads to the reduction of CD44 surface expression and HA levels, being both parameters key factors in the mechanism of action of imatinib.

## Materials and Methods

### Reagents

Recombinant high molecular weight (HMW, 1.5–1.8 × 10^6^ Da) HA (CPN spol.s.r.o Czech Republic) was supplied by Farmatrade (Argentina). The Km81 anti-CD44 monoclonal Ab (MAb) was kindly provided by Dr. Pauline Johnson (University of British Columbia, Vancouver, Canada). bHABP, 4MU, X-gal and 4′,6-diamidino-2-phenylindole (DAPI) were purchased from Sigma-Aldrich (USA). Drugs used were imatinib (Novartis, Switzerland) and vincristine (VCR, Filaxis, Argentina). Antibodies (Ab) against Abl (K-12: sc-131), pAkt1/2/3 (Thr308-R, sc-16646-R), Akt1/2/3 (H136, sc-8312), pERK (Tyr204-R, sc-101761), ERK (C14, sc154), and β-actin (C11, sc-1615), horseradish peroxidase-labeled anti-rabbit (sc-2030) and anti-goat (sc-2033) secondary antibodies, and the biotinylated anti-rat (sc-2041) secondary antibody were purchased from Santa Cruz Biotechnology (USA). The avidin-PE was purchased from eBioscience (USA). The [^3^H]TdR was purchased from Perkin-Elmer (Boston, USA). RPMI 1640, L-glutamine, streptomycin and penicillin were purchased from Invitrogen (Argentina). The Annexin-V-PE Apoptosis Detection Kit I was purchased from BD Pharmingen™ (BD Bioscience, USA).

### Cell culture

Human CML cell lines K562 and Kv562 were grown in suspension cultures at 37 °C in a 5% CO_2_ atmosphere with RPMI 1640 supplemented with 10% heat inactivated fetal bovine serum (FBS), 2 mM L-glutamine, 10 mM HEPES buffer, 5 × 10^−5^ M 2-mercaptoethanol, 100 µg/ml streptomycin and 100 IU/ml penicillin (RPMI-C). Kv562 cell line was cultured in the presence of 150 ng/ml (162 nM) VCR^19^. Kv562 cells have been obtained by incubating K562 cells with increasing doses of VCR. In these cells, resistance developed as a result of both P-glycoprotein, Pgp, and PI3K over-activation^19,21^, with these mechanisms also being involved in imatinib resistance^48,52^.

#### Measurement of HA levels by enzyme-linked immunosorbent assay

A suspension containing 5 × 10^5^ cells was grown for 72 h, as described for cell culture, in the presence of either RPMI-C alone, imatinib (0.25, 0.5 or 2 μM) or 4MU (500 or 100 μM). HA levels were measured in the cell supernatant by a competitive enzyme-linked immunosorbent assay (ELISA), as described previously^53^. Briefly, 96 well microtiter plates were coated with 100 µg/mL HMW-HA at 4 °C. Samples and standard HMW-HA were incubated with 0.75 µg/mL bHABP at 37 °C. Unoccupied sites were blocked and incubated with samples at 37 °C for 4 h. The bHABP bound was determined with an avidin–biotin detection system. Sample concentrations were derived from a standard curve.

### CD44 expression by flow cytometry

After treating cells with either RPMI-C (control) or imatinib (0.25, 0.5 or 2 μM) for 24 h, cells were fixed with PBS plus 2% PFA for 15 min. After that, cells were permeated or not with PBS plus 0.2% Triton X-100 for 30 min. Cells were then blocked with phosphate-buffered saline (PBS) containing 2% normal human serum for 30 min at 4 °C. After incubation, anti-CD44 Km81 was added followed by the addition of a biotinylated secondary Ab and Av-PE. All incubations were carried out for 1 h at 4 °C in the dark and with washes with cold PBS in between. Stained cells were acquired on a Pas III flow cytometer (Partec, Germany) and analyzed with the WinMDI 2.8 software (Scripps Institute, La Jolla)^53^. The corresponding isotypic controls were included in each assay. The ATCC U937 cell line was grown under the same conditions as described above and used as Ph chromosome negative control.

### Cell proliferation

Cell proliferation was analyzed by the [^3^H]TdR uptake assay evaluated at 48 h in 96-well microtiter plates^19^. Fifty thousand cells per well (2.5 × 10^5^ cells/ml) were used. Cells were grown at 37 °C in a 5% CO_2_ atmosphere with either RPMI-C, HA (300 µg/ml) or 4MU (100 μM) or imatinib (0.25; 0.5 or 2 µM), or combinations of them. After pulsing with 1 μCi [^3^H]TdR for 6 h, cells were harvested and counted in a liquid scintillation counter (Beckman, MD). Results were calculated from the mean cpm of [^3^H]TdR incorporated in triplicate cultures. Untreated cells represented 100% cell survival. Cell viability at the beginning of the experiment was higher than 95%, as assessed by the Trypan blue dye.

#### Evaluation of apoptosis

Cells (5 × 10^5^ cells/ml) were treated with either RPMI-C or HA (300 µg/ml) or 4MU (100 μM) or imatinib (0.25; 0.5 or 2 µM µM), or combinations of them for 48 h at 37 °C in a 5% CO_2_ atmosphere. Then, membrane asymmetry, DNA fragmentation and the hypodiploid DNA content were evaluated. For membrane asymmetry, the Annexin-V-PE Apoptosis Detection Kit I (BD Biosciences, USA) was used following the manufacturer’s instructions. A PAS III flow cytometer (FC) (Partec, Germany) was used to acquire data, which were analyzed with the WinMDI 2.8 software (Scripps Institute, La Jolla, USA). For DNA fragmentation and the hypodiploid DNA content, cells were fixed with phosphate buffer saline (PBS) plus 2% *p-*formaldehyde (PFA) or 70% ethanol, respectively, stained with DAPI and evaluated by fluorescence microscopy (Olympus BX51, America Inc.) and FC (PAS III flow cytometer, Partec Germany), respectively^54,55^.

### Evaluation of senescence

Cell senescence was assessed through SA-β-gal activity and the presence SAHF^56^. For SA-β-gal, cells (5 × 10^5^ cells/ml) were incubated with either RPMI-C, HA (300 µg/ml) or 4MU (100 μM) or imatinib (0.25; 0.5 or 2 µM), or combinations of them at 37 °C in a 5% CO_2_ atmosphere. After 48 h, cells were fixed with PBS plus 2% PFA and washed with PBS. Each vial was incubated for 24 h at 37 °C with 1 ml of staining solution (1 mg/ml X-gal, 5 mM potassium ferricyanide, 5 mM potassium ferrocyanide, 2 mM MgCl_2_, 150 mM NaCl, 30 mM citric acid/phosphate pH = 6). Cells were washed twice with PBS and the SA-β-gal activity was evaluated in an Olympus BX51 (America Inc.) microscope. Blue cells were considered positive. For each condition, 200 cells were counted and the percentage of SA-β-gal positive cells was calculated^25,56^. For SAHF, cells were subjected to similar treatments. After 48 h, cells were fixed with PBS plus 2% PFA, washed and incubated with 1 μg/ml DAPI in PBS plus 0.2% Triton X-100 for 30 min at room temperature. Cells were analyzed by fluorescence microscopy (Olympus BX51, America Inc.).

### Western blot

Cells were treated with either RPMI-C, HA (300 µg/ml) or 4MU (100 μM) or imatinib (0.5 or 2 µM) or combinations of them for 24 h at 37 °C in a 5% CO_2_ atmosphere. Cells were then lysed with hypotonic buffer (0.02 M Tris pH = 8, 0.15 M NaCl, 0.1 M NaF, 1 mM PMSF, 10% glycerol, 1% NP-40, 20 µg/ml leupeptin, 20 µg/ml aprotinin, 1 mM sodium *o-*vanadate). After centrifugation (13000 rpm 30 min), equal amounts of protein were resolved by SDS-polyacrylamide gel electrophoresis and transferred onto a PVDF membrane (Osmonics Inc., Gloucester, MA). The membrane was blocked and incubated with specific antibodies to Abl, p-Akt, Akt, p-ERK, ERK or β-actin overnight at 4 °C followed by incubation with a horseradish peroxidase-labeled secondary antibody for 2 h at 37 °C. The reaction was developed with a chemiluminescent detection system. Gel images obtained with a digital camera were subjected to densitometric analysis with the Image Scion Software (Scion Corporation, USA)^19,57^. Akt and ERK were evaluated after stripping on the membranes on which pAkt and pERK had previously been determined, respectively.

### Statistical analysis

All results were analyzed by one way-ANOVA and the Bonferroni’s test. Analyses were performed with the Prism software (Graph Pad, San Diego, CA, USA). P values < 0.05 were regarded as statistically significant^19^. The drug combination analysis was performed according to the Bliss independence model^58^.

## Supplementary information

Supplementary data

## Data Availability

Data generated or analyzed during this study are available from the corresponding author on reasonable request.
