# Author Correction: Hyaluronan abrogates imatinib-induced senescence in chronic myeloid leukemia cell lines

**DOI:** 10.1038/s41598-020-69087-8

**Published:** 2020-07-16

**Authors:** Silvina Lompardía, Mariángeles Díaz, Matías Pibuel, Daniela Papademetrio, Daniela Poodts, Cintia Mihalez, Élida Álvarez, Silvia Hajos

**Affiliations:** 1 Departamento de Microbiología, Inmunología Y Biotecnología, Cátedra de Inmunología, Universidad de Buenos Aires, Facultad de Farmacia Y Bioquímica, Buenos Aires, Argentina; 2 CONICET, Instituto de Estudios de La Inmunidad Humoral (IDEHU), Universidad de Buenos Aires, Buenos Aires, Argentina

Correction to: *Scientific Reports* 10.1038/s41598-019-47248-8, published online 29 July 2019

This Article contains an error. In Figure 3A, the image used for the Kv562 untreated condition is incorrect. The correct panel of Figure 3A appears below as Figure 1.

**Figure 1:**
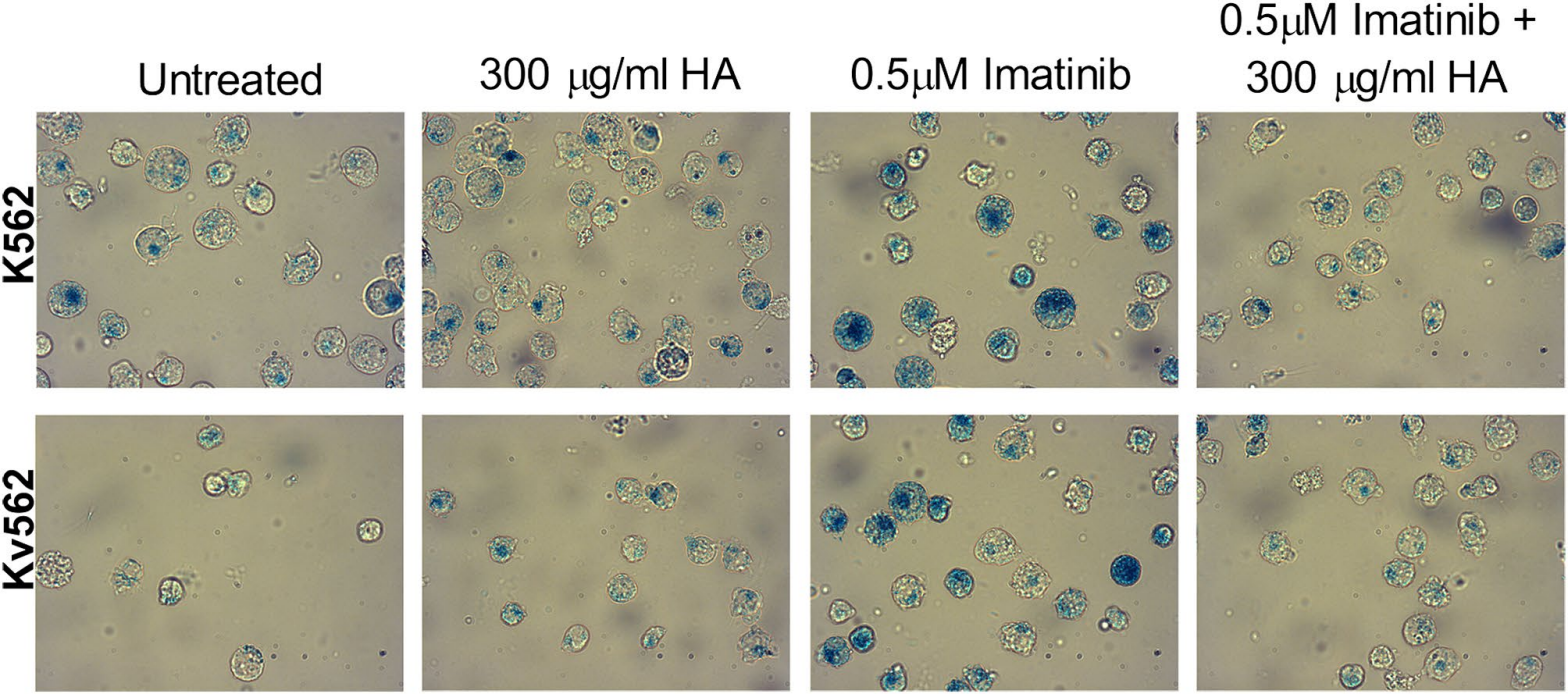
.

