# Supplementary material for: Hyaluronan abrogates imatinib-induced senescence in chronic myeloid leukemia cell lines
Source: Sci Rep. 2019 Jul 29;9:10930. doi: 10.1038/s41598-019-47248-8 (PMC6662747; doi:10.1038/s41598-019-47248-8)
Supplement: Supplementary file 1 — Supplementary data [file 41598_2019_47248_MOESM1_ESM.pdf]

Hyaluronan abrogates imatinib-induced senescence in chronic myeloid leukemia cell lines

Lompardía, Silvina[1,2*†]; Díaz, Mariángeles[2†]; Pibuel, Matías[1,2]; Papademetrio, Daniela[1,2]; Poodts, Daniela[1]; Mihalez, Cintia[1,2]; Álvarez, Élida[1,2]; Hajos, Silvia[1,2].

[1] Universidad de Buenos Aires. Facultad de Farmacia y Bioquímica. Departamento de Microbiología, Inmunología y Biotecnología. Cátedra de Inmunología. Buenos Aires, Argentina.

[2] Universidad de Buenos Aires. CONICET. Instituto de Estudios de la Inmunidad Humoral (IDEHU). Buenos Aires, Argentina.

* SL: sil.lompardia@gmail.com

[†]SL and MD contributed equally to the realization of this work.

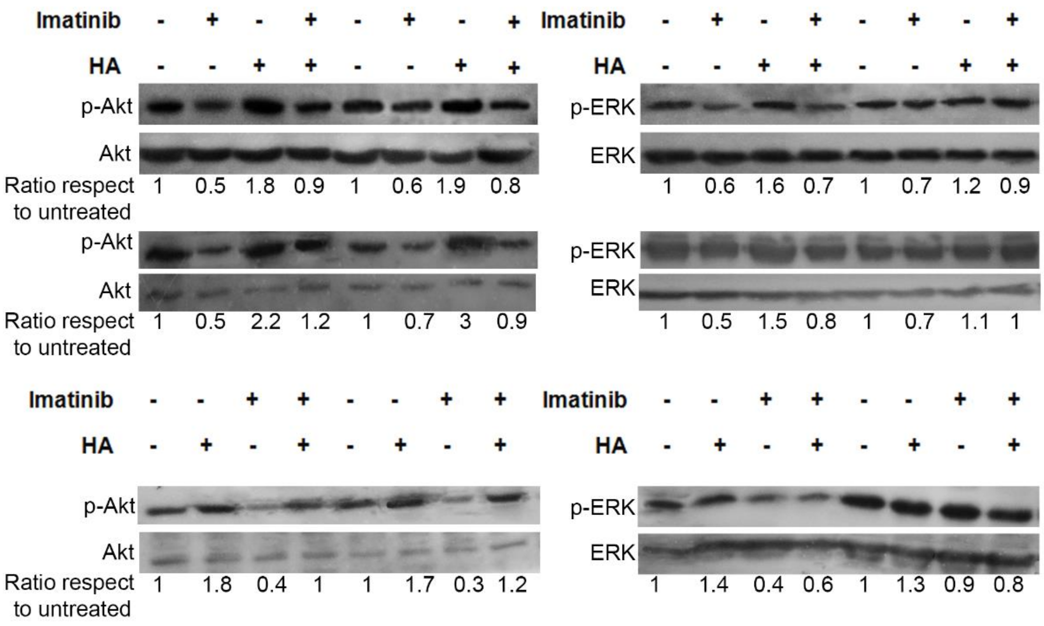

Supplementary **Fig S1**. Biological replicates of WB assays. Cells were treated either with imatinib (0.5µM for K562 and 2µM for Kv562 cells), 300 µg/ml HA or a combination of both for 24 h. The phosphorylation of Akt and ERK was evaluated by WB. Each experiment was performed at least five times independently. The ratio was calculated as:

Ratio= $[(pAkt/\beta\text{-actin}) / (Akt/\beta\text{-actin})]_{treated} / [(pAkt/\beta\text{-actin}) / (Akt/\beta\text{-actin})]_{untreated}$

Ratio= $[(pERK/\beta\text{-actin}) / (ERK/\beta\text{-actin})]_{treated} / [(pERK/\beta\text{-actin}) / (ERK/\beta\text{-actin})]_{untreated}$

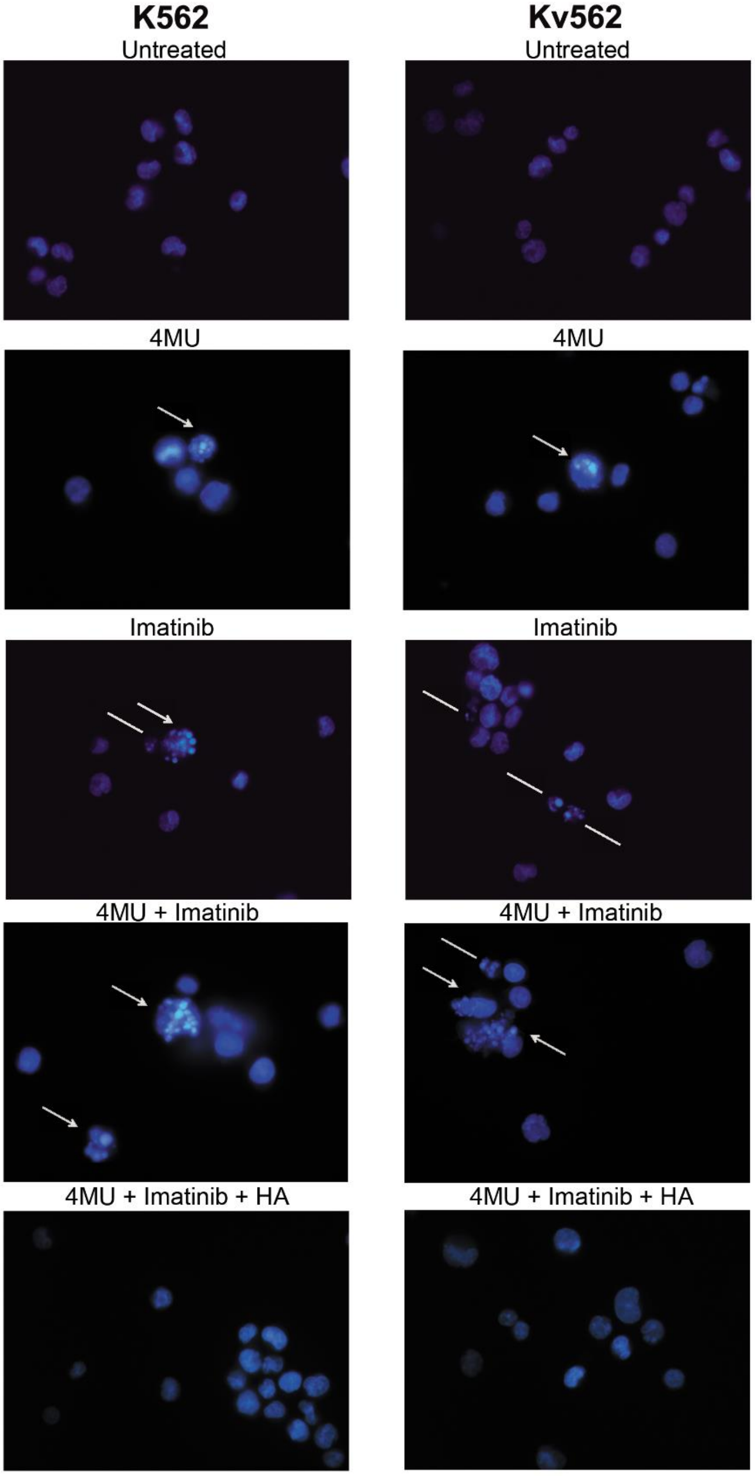

Supplementary **Fig. S2**. Evaluation of SAHF. Cells were treated either with imatinib (0.5µM for K562 and 2 µM for Kv562 cells), 100 µM 4MU, the combination of both, or the combination of both plus 300 µg/ml HA for 48 h. The presence of SAHF was evaluated by fluorescence microscopy using DAPI stain (magnification 400X). Arrows indicate nuclei with SAHF, while lines indicate nuclei with DNA fragmentation, which are characteristic of senescent and apoptotic cells, respectively.
